# Molecular Networks Governing Plant Responses to Heat and Cold Stress

**DOI:** 10.3390/plants14132073

**Published:** 2025-07-07

**Authors:** Ran Zhang, Lin Yang, Huan Zhang, Yingyu Yang, Lu Wen, Aoran Yin, Liwen Fu

**Affiliations:** 1 Shanghai Collaborative Innovation Center of Agri-Seeds, School of Agriculture and Biology, Shanghai Jiao Tong University, Shanghai 200240, China; zr_807@sjtu.edu.cn (R.Z.); yanglin2023@sjtu.edu.cn (L.Y.); zhang_h@sjtu.edu.cn (H.Z.); yangyingyu@sjtu.edu.cn (Y.Y.); wenlu0902@sjtu.edu.cn (L.W.); yarrtata@sjtu.edu.cn (A.Y.); 2 Joint Center for Single Cell Biology, School of Agriculture and Biology, Shanghai Jiao Tong University, Shanghai 200240, China

**Keywords:** heat stress responses, cold stress responses, phytohormone, unfolded protein response

## Abstract

Global warming is leading to an increase in extreme-temperature events, posing a significant threat to crop productivity and global food security. Plants have evolved sophisticated mechanisms to perceive and respond to both heat and cold stress. While these mechanisms share certain similarities, they also exhibit distinct differences, enabling plants to effectively cope with extreme temperatures. This review summarizes recent findings on the mechanisms underlying plant perception and response to extreme temperature stresses. Additionally, we compare the signaling pathways for heat and cold stress in plants and discuss the remaining challenges in the field. Finally, we address unresolved issues and propose future directions.

## 1. Introduction

The increasing frequency of extreme-temperature events, a consequence of ongoing global warming, poses a significant threat to crop production and, consequently, to global food security [1,2]. For instance, studies indicate that for every 1 °C increase in average temperature, yields of major crops are projected to decline, with wheat decreasing by 6.0%, corn by 7.4%, rice by 3.2%, and soybeans by 3.1% on average [3]. Furthermore, low-temperature stress threatens over 15 million hectares of rice cultivation areas across 24 countries, including major producers such as China, Japan, South Korea, and India, as well as regions within Australia, particularly during critical growth stages [4].

Sessile plants have evolved a sophisticated signaling network to perceive and respond to extreme temperatures, a capacity fundamental for their survival [3]. Plants can rapidly sense both extreme high and low temperatures [5,6]. Interestingly, while plants employ distinct mechanisms to sense heat or cold stress, they also share common features. Membrane fluidity serves as a key element in sensing both types of temperature extremes. Temperature fluctuations dramatically alter plasma membrane fluidity, consequently affecting the activity of membrane-localized proteins [5,6]. This, in turn, activates Ca^2+^ signaling or modifies lipid composition, initiating downstream signaling cascades. In addition to these shared mechanisms, plants also possess specific pathways for perceiving heat or cold stress. Upon the perception of heat or cold signals, plants initiate a multitude of downstream responses to mitigate the stress. Transcriptional regulation plays a crucial role in both heat and cold stress responses. In heat stress, HsfA1s function as master regulators, orchestrating the transcriptional regulation of heat stress response genes [7]. Conversely, CBF1 serves as the central component in plant cold response pathways [3,5]. Both HsfA1s and CBF1 are subject to intricate upstream regulation, allowing for the fine-tuning of the plant’s response to heat or cold stress through transcriptional regulation [5,6].

In addition to the two master regulators involved in the transcriptional regulation of heat and cold responses, various phytohormones also play critical roles in plant responses to extreme-temperature stresses, thereby adding to the complexity and sophistication of the signaling network.

This review summarizes recent findings on the mechanisms by which plants perceive heat and cold stress signals and their subsequent responses to extreme temperatures. We then compare the heat and cold signaling networks, addressing current unresolved issues and proposing potential future research directions.

## 2. Results

### 2.1. Key Temperature Pathways in Plants

Temperature is a crucial environmental factor influencing plant growth and development. Under varying conditions, plants employ distinct signaling networks to perceive and respond to temperature signals. Generally, there are four major temperature response pathways in plants.

#### 2.1.1. Thermomorphogenesis

Thermomorphogenesis, the adaptation of plant morphology and development to warm temperatures, is a widespread phenomenon that varies among different plant species. In *Arabidopsis thaliana*, a model organism, thermomorphogenic responses are typically observed within a temperature range of 22 to 28 °C. These responses include a series of morphological changes, such as the elongation of hypocotyls, roots, stems, and petioles, as well as upward leaf bending and a reduction in leaf blade size [6,8]. These responses result in an open rosette structure that enhances efficient leaf cooling [6,8]. A central regulator of *Arabidopsis* thermomorphogenesis is the PHYTOCHROME-INTERACTING FACTOR 4 (PIF4) transcription factor [6,8]. PIF4 is regulated both transcriptionally and post-translationally by temperature and orchestrates the reprogramming of the transcriptome in response to elevated ambient temperatures [6,8].

#### 2.1.2. Heat Stress

In all plants, temperatures exceeding the optimal range (above 30 °C for *Arabidopsis*) trigger heat stress. This stress adversely affects seed germination, photosynthesis, water use efficiency, cell proliferation, flowering, pollen viability, and overall plant productivity [6,8]. Plants have developed a sophisticated signaling network to detect and respond to heat stress.

#### 2.1.3. Vernalization

Vernalization is a biological process wherein certain plants require extended exposure to cold temperatures to initiate or accelerate their transition from vegetative growth to the flowering stage. This adaptation ensures that flowering occurs at an optimal time, typically following winter, to coincide with favorable environmental conditions for reproduction. During this process, plants must undergo a prolonged period of low temperatures (typically 1–10 °C) lasting from weeks to months, depending on the species. Exposure to cold modifies gene expression, often via epigenetic mechanisms such as histone modification. For instance, in *Arabidopsis thaliana*, cold exposure silences the *FLC* (*FLOWERING LOCUS C*) gene, which normally inhibits flowering [9].

#### 2.1.4. Cold Stress

Low-temperature stress, a significant abiotic factor, considerably impacts crop production and the geographical distribution of plant species [5]. Plants are typically exposed to two distinct forms of this stress: chilling, which occurs at temperatures between 0 and 15 °C, and freezing, which occurs at temperatures below 0 °C [8]. Notably, when subjected to chilling temperatures, certain plants can undergo a process of cold acclimation [8]. Cold acclimation refers to the phenomenon whereby prior exposure to moderately suboptimal, chilling temperatures enhances a plant’s subsequent tolerance to more severe freezing stress [8]. Plants have also evolved a sophisticated signaling network to perceive and respond to cold stress.

This review focuses on plant sensing and response mechanisms to heat and cold stress.

### 2.2. Thermosensing Under Heat Stress

#### 2.2.1. Thermosensing at the Cell Wall

In plant cells, the cell wall acts as the primary interface and initial barrier encountered under heat stress. Consequently, heat stress induces significant alterations in cell wall composition and structure, with cell wall remodeling serving as a critical mechanism for heat stress perception [6,10,11]. Specifically, heat stress activates pectin methylesterase (PME, EC 3.1.1.11), a key cell wall remodeling enzyme [6,10,11]. Upon activation, PME catalyzes the demethylesterification of pectin, releasing protons and consequently reducing the cell wall pH. This reduction in pH subsequently promotes the activity of endopolygalacturonase (PG), leading to cell wall loosening. This loosening facilitates Ca^2+^ influx from the apoplast into the cytoplasm, initiating downstream heat stress responses (HSRs) [6,10,11].

#### 2.2.2. Thermosensing at the Plasma Membrane

The plasma membrane inherently functions as a thermosensor due to the temperature sensitivity of its fluidity [12,13]. Elevated temperatures induce significant alterations in membrane lipid composition, disrupting the ordered arrangement of fatty acids and consequently affecting the rotational and lateral diffusion of lipids, as well as the packing density of head groups within the bilayer. These collective changes, characterized as alterations in membrane fluidity, trigger a cascade of effects, directly or indirectly influencing the folding, mobility, and activity of membrane proteins [6,8]. Indeed, several membrane proteins have been identified as putative thermosensors. Plasma membrane-localized CYCLIC NUCLEOTIDE-GATED Ca^2+^ CHANNELS (CNGCs) are crucial regulators of heat-triggered Ca^2+^ influx in plants [14]. Initial evidence for the involvement of CNGCs in the plant HSR stemmed from studies on *Arabidopsis cngc2* and *Physcomitrella patens cngcb null* mutants [15]. Specifically, loss-of-function mutations in *CNGC2*/*CNGCb* conferred enhanced thermotolerance in the seedling stage [15]. However, the role of *At*CNGC2 appears to be developmentally regulated, as its dysfunction was correlated with increased thermosensitivity during the reproductive stage [16]. Additionally, *At*CNGC4, *At*CNGC6, and *At*CNGC16 in *Arabidopsis* have also been implicated in heat-stress adaptation [17,18]. Similarly, in rice, *Os*CNGC14 and *Os*CNGC16 function as temperature switches that promote tolerance in both heat and chilling stress [19]. The activation mechanisms of these CNGCs are closely associated with cyclic nucleotides. Cyclic AMP (cAMP) and cyclic guanosine monophosphate (cGMP) are activating molecules that activate CNGCs through direct binding [20,21]. Specifically, in *Arabidopsis*, CNGC6 is activated by cAMP and promotes the expression of heat shock protein (HSP) genes [18]. Therefore, it is possible that cAMP/cGMP producers such as adenylyl/guanylate cyclase or the Toll/interleukin-1 receptor function upstream of CNGCs and serve as putative thermosensors [6]. Beyond CNGCs, other plasma membrane-localized proteins are also crucial in heat stress perception. RESPIRATORY BURST OXIDASE HOMOLOG D (RBOHD), a plasma membrane-localized NADPH oxidase, is a primary producer of reactive oxygen species (ROS) in response to hormonal and environmental cues [22,23]. High temperature activates RBOHD and results in the accumulation of ROS [6]. Annexin 1 (ANN1), another plasma membrane-localized protein, functions downstream of RBOHD by detecting ROS and facilitating Ca^2+^ influx [24]. Such elevated Ca^2+^ levels may subsequently activate RBOHD in turn, thereby forming self-propagating Ca^2+^/ROS waves [6].

#### 2.2.3. Thermosensing via Lipids

Apart from Ca^2+^ and ROS, lipids also act as signaling molecules, translating environmental cues into intracellular responses. Lipids, including PHOSPHATIDIC ACID (PA) and PHOSPHATIDYLINOSITOL 4,5-BISPHOSPHATE (PIP2), along with their metabolic enzymes, such as phospholipase C and D (PLC/PLD), diacylglycerol kinase (DGK), and PHOSPHATIDYLINOSITOL 4-PHOSPHATE 5-KINASE (PIP5K), play diverse and critical roles in regulating cellular responses to environmental stresses [25]. In *Arabidopsis* seedlings, heat stress triggers an increase in PA and PIP2 abundance within 2 min [25]. PLD and PLC/DGK pathways produce PA, exhibiting a rapid response to heat stress with dynamic fluctuations within minutes [25]. The PA produced by these pathways can then destabilize the plasma membrane, leading to changes in the activity of membrane proteins [26]. Furthermore, PA can bind to downstream proteins to mediate stress responses [27,28]. When exposed to high temperature, HS increases the content of Ca^2+^ and H_2_O_2_; PLD is activated by elevated Ca^2+^ and H_2_O_2_ levels, suggesting PLD functions as a downstream component of thermosensing [13,29]. When PLCs break down PIP2 and PHOSPHATIDYLINOSITOL 4-PHOSPHATE (PI4P), they yield two key products: DAG and INOSITOL-1,4,5-TRISPHOSPHATE (IP3). The IP3 then acts within the cytoplasm, prompting the release of Ca^2+^ from internal storage sites. This process not only localizes PLCs to the membrane but may also equip them with a role in detecting temperature changes [30].

#### 2.2.4. Thermosensing Through Liquid–Liquid Phase Separation (LLPS)

Heat stress triggers a global reduction in protein synthesis, leading to the formation of stress granules (SGs) via LLPS. This process also serves as a crucial heat stress sensing mechanism. Specifically, in *Arabidopsis*, RNA-binding glycine-rich proteins RBGD2 and RBGD4 condense into heat-induced SGs, where they interact with specific proteins and transcripts, ultimately initiating heat stress responses [31]. Furthermore, ALBA proteins ALBA4, ALBA5, and ALBA6 (ACETYLATION LOWERS BINDING AFFINITY 4, 5, and 6), also undergo phase separation into both SGs and processing bodies (P-bodies) under heat stress [32]. These ALBA proteins directly bind to selected messenger RNAs (mRNAs), including heat stress transcription factor (HSF) mRNAs, and sequester them within SGs and PBs, thereby protecting them from degradation during heat stress in *Arabidopsis* [32]. Furthermore, high temperature enhances the LLPS of GRP7 (GLYCINE-RICH RNA-BINDING PROTEIN 7), driving the formation of SGs, which facilitates RNA assembly and sequesters the translation initiation factor EUKARYOTIC INITIATION FACTOR 4E1 (eIF4E1) and the mRNA chaperones CSP1 and CSP3 (COLD SHOCK PROTEIN 1 and 3), thereby inhibiting mRNA translation and, by reducing the synthesis of aberrant proteins, ultimately enhancing plant tolerance to heat stress [33].

#### 2.2.5. Thermosensing via Protein Translocation

Heat stress triggers alterations in the subcellular localization of proteins, serving as a crucial mechanism for sensing and responding to heat stress signals. The nucleus is a common destination for these translocating proteins. For instance, in *Arabidopsis*, the cytoplasmic glycolytic enzyme GLYCERALDEHYDE-3-PHOSPHATE DEHYDROGENASE (GAPC) translocates to the nucleus upon heat stress, where it interacts with and activates the transcription factor NUCLEAR FACTOR Y SUBUNIT C10 (NF-YC10), thereby facilitating signaling transmission in response to excessive heat exposure [34]. Similarly, sumoylated ARABIDOPSIS B-CELL LYMPHOMA2 (BCL-2)-ASSOCIATED ATHANOGENE 7 (*At*BAG7) translocates from the endoplasmic reticulum (ER) to the nucleus under heat stress, interacting with the WRKY29 transcription factor [35]. Moreover, transcription factors are proteins that bind to specific DNA sequences to regulate the transcription of genetic information into RNA, a process that occurs in the nucleus. Under heat stress, some transcription factors undergo relocalization to the nucleus to fulfill their regulatory functions. For instance, in rice, the heat stress-induced translocation of NAM, ATAF AND CUC 23 (*Os*NAC23) transcription factor to the nucleus may represent a mechanism for its functional activation, complementing transcriptional regulation under stress conditions [36]. Furthermore, high temperature induces the translocation of *Os*NTL3 (a member of NAC transcription factors) from the membrane to the nucleus, eliciting downstream heat stress responses [37]. However, protein translocation is not limited to the nucleus. For example, research on Thermo-Tolerance 3 (TT3) in rice has shown that the plasma membrane-localized E3 ligase TT3.1 translocates to endosomes under heat stress [38].

### 2.3. HSR in Plants

#### 2.3.1. HsfA1-Mediated Transcriptional Response to Heat Stress

CLASS A1 HEAT SHOCK FACTOR (HsfA1) proteins are master regulators of the transcriptional response to heat stress. In *Arabidopsis*, the HsfA1 family comprises four members: HsfA1a, b, d, and e [7]. The quadruple mutant of these *HsfA1* genes exhibits hypersensitivity to heat stress, highlighting their crucial role in heat stress responses [7]. HsfA1s’ activity is tightly controlled through multiple regulatory mechanisms. At the transcriptional level, HsfA1 genes are transcriptionally activated by heat stress [3,39]. At the post-translational level, HsfA1s’ activity and subcellular localization are intricately regulated. Under normal conditions, the HSP70/90 complex interacts with HsfA1s, inhibiting their transcription factor activity and preventing their nuclear localization. However, during heat stress, this repression by HSP70/90 is relieved, allowing HsfA1s to translocate to the nucleus and initiate the expression of heat stress-responsive genes [3,40,41]. Post-translational modifications, such as phosphorylation, can further modulate HsfA1s’ function. CDC2a, a cyclin-dependent kinase, phosphorylates HsfA1d, thereby inhibiting its DNA-binding activity [42]. Heat-induced cytosolic Ca^2+^ elevation is perceived and decoded by calmodulin (CaM), which activates CBK3 through direct interaction [43]. CBK3 then phosphorylates HsfA1s, inducing their activity and promoting the expression of heat stress-responsive genes [43]. Meanwhile, CaM3 also interacts with CALMODULIN-BINDING PROTEIN PHOSPHATASE 7 (PP7), a CaM-binding phosphatase, which also interacts with HsfA1 and may enhance HsfA1s’ activity through dephosphorylating it at a different site [44]. Brassinosteroids (BRs) positively regulate thermotolerance in *Arabidopsis*. Mechanistically, in the absence of BR signaling, activated BRASSINOSTEROID INSENSITIVE2 (BIN2) interacts with and phosphorylates HsfA1d. This phosphorylation diminishes both the nuclear localization and the DNA-binding affinity of HsfA1d, thereby compromising its function. Conversely, the activation of BR signaling results in a significant inhibition of BIN2 activity, which restores the nuclear translocation and DNA-binding ability of HsfA1d, ultimately activating the heat stress responses [45]. Finally, the protein stability of HsfA1 may also be subject to regulation. A recent study demonstrated that the co-chaperone protein, HSP70-HSP90 organizing protein (HOP), interacts with and promotes the stability of HsfA1a under warm temperatures [46]. However, it remains unclear whether this mechanism also functions under heat stress conditions, representing a gap in current research.

Following activation by heat stress, HsfA1s mediates heat stress-responsive genes and microRNAs to regulate heat tolerance. DEHYDRATION-RESPONSIVE ELEMENT BINDING PROTEIN 2A (DREB2A) is a key transcription factor promoting heat tolerance, functioning downstream of HsfA1s [3,6,47,48,49]. At the transcriptional level, under normal conditions, GROWTH REGULATING FACTOR 7 (GRF7) directly binds to the promoter region of *DREB2A* and represses its expression [50]. However, under heat stress, HsfA1s, JUNGBRUNNEN 1 (JUB1), and MULTIPROTEIN BRIDGING FACTOR 1C (MBF1c) promote the expression of *DREB2A* [51,52,53]. At the post-translational level, DREB2A also undergoes sophisticated regulation. Under normal conditions, CASEIN KINASE 1 (CK1)-mediated phosphorylation targets DREB2A for degradation. Heat stress reduces this phosphorylation, thereby increasing DREB2A protein abundance [3,6]. Furthermore, the heat-induced degradation of RADICAL-INDUCED CELL DEATH 1 (RCD1) releases its destabilizing effect on DREB2A [3,54]. In addition to DREB2A, HsfA1s also induce the expression of *HsfA2* [3,6]. *HsfA2* expression is also induced by ethylene signaling via the ETHYLENE INSENSITIVE3-ETHYLENE RESPONSE FACTOR95/97 (EIN3-ERF95/97) cascades [55]. The post-translational modification of HsfA2 also occurs via sumoylation. In *Arabidopsis*, SUMO1 interacts with the transcription factor HsfA2, reducing HsfA2′s transcriptional activity [56,57]. Meanwhile, HsfA2 plays a crucial role in the epigenetic regulation of heat stress responses. H3K4 di-/tri-methylation (H3K4me2/me3) marks regions of recent transcriptional activity at specific loci, such as *HSP22.0* and *APX2*. These chromatin modifications persist beyond the initial HSR and facilitate transcriptional memory, enabling hyper-induction upon recurring stress. HsfA2, which transiently binds to target promoters following heat stress, plays a crucial role in maintaining H3K4 methylation and sustaining gene expression [58]. HsfA1b induces miR398 expression, which inhibits the expression of ROS scavenger genes CSD1 (Cu/Zn SOD SUPEROXIDE DISMUTASE 1), CSD2 (Cu/Zn SOD SUPEROXIDE DISMUTASE 2), and CCS1 (COPPER CHAPERON FOR SUPEROXIDE DISMUTASE 1). This inhibition results in ROS overproduction, subsequently activating HsfA1 [3,59].

#### 2.3.2. UPR and HSR

Extreme heat stress induces protein misfolding, leading to an increased burden of misfolded or unfolded proteins in the ER, a condition that results in ER stress in plants. This subsequently activates the unfolded protein response (UPR), a key regulatory mechanism also implicated in the heat stress response [60]. The UPR activates stress-response genes by inducing the translocation of ER membrane-bound bZIP (BASIC LEUCINE ZIPPER) transcription factors (bZIP28 and bZIP60) to the nucleus via distinct mechanisms [61,62]. Under normal conditions, the ER-localized chaperone BiP interacts with bZIP28, retaining it within the ER. However, upon the induction of ER stress, bZIP28 undergoes translocation from the ER to the Golgi apparatus, a process mediated by its lumen-facing C terminus domain [63]. Subsequently, in the Golgi, bZIP28 is sequentially processed by SITE 1 PROTEASE (S1P) and SITE 2 PROTEASE (S2P) [63]. This proteolytic activation enables the processed bZIP28 to translocate to the nucleus, where it functions to activate the heat shock response [63]. In response to the heat stress-induced UPR, INOSITOL-REQUIRING ENZYME 1 (IRE1) initiates the splicing of *bZIP60* mRNA [62]. This process involves the cleavage of a 23-base-pair segment from the *bZIP60* mRNA transcript, resulting in a spliced mRNA molecule [62]. The translation of this spliced mRNA produces a truncated bZIP60 protein, which critically lacks the transmembrane domain [62]. Consequently, this truncated bZIP60 protein is able to translocate to the nucleus, where it functions as a transcription factor, activating the expression of genes associated with the UPR [62]. Besides the bZIP transcription factors, NAC (NAM, ATAF1/2, AND CUC2) TRANSCRIPTION FACTORS (NTLs) have also been shown to mediate the UPR during heat stress [3,37]. *Os*NTL3, a member of the rice NTL transcription factor family, is a membrane-associated NTL that relocates to the nucleus under heat and ER stresses [37]. *Os*NTL3 activates genes encoding *HSPs* and genes involved in ER protein folding, thereby conferring thermotolerance in rice [37].

#### 2.3.3. Semiautonomous Organelles and HSR

Semiautonomous organelles, including mitochondria and chloroplasts, are also critical components of heat stress tolerance [6]. Heat stress can induce genome rearrangement within mitochondria [6]. This rearrangement is repressed by two mitochondrial proteins, MUTS HOMOLOG 1 (MSH1) and RECOMBINASE A3 (RECA3), both of which are encoded by nuclear genes. Notably, MSH1 and RECA3 negatively regulate thermotolerance [64]. Mitochondria are essential organelles responsible for ATP generation via OXIDATIVE PHOSPHORYLATION (OXPHOS). They also produce ROS, which function as both signaling molecules and damaging agents. MITOCHONDRIAL TRANSCRIPTION TERMINATION FACTOR-RELATED (mTERF) proteins regulate organelle gene expression through interactions with organellar DNA or RNA. For instance, mTERF18/SHOT1 (SUPPRESSOR OF HOT1-4 1) enables plants to better tolerate heat and oxidative stresses, presumably by reducing ROS production and oxidative damage. Interestingly, SHOT1 interacts with three homologs of the animal ATPase family AAA DOMAIN-CONTAINING PROTEIN 3 (ATAD3), a protein implicated in mitochondrial nucleoid organization. Consistent with the *shot1* phenotype, the disruption of ATAD3 function leads to nucleoid disorganization, the reduced accumulation of complex I, and enhanced heat tolerance [65].

Chloroplasts also play a crucial role in plant heat stress responses. In *Arabidopsis*, heat stress significantly alters the chloroplast transcriptome, disrupting the photosynthetic system [66]. In rice, thermotolerance is modulated by two proteins, TT3.1 and TT3.2, with opposing functions. Specifically, TT3.1 acts as a positive regulator, whereas TT3.2, a chloroplast precursor protein, functions as a negative regulator [38]. Under heat stress, TT3.1, which exhibits high ligase activity, is sorted into endosomes. Within these endosomes, TT3.1 interacts with TT3.2 precursor proteins, facilitating their ubiquitination. The ubiquitinated TT3.2 is then delivered to endosomes for vacuolar degradation. This degradation process reduces the amount of TT3.2 imported into chloroplasts. Consequently, the disruption of the PSII complex and thylakoids is prevented, leading to enhanced thermotolerance and, ultimately, increased rice productivity [38]. Small heat shock proteins and molecular chaperones are also functional components within chloroplasts. For example, the chloroplast small heat shock protein HSP21 interacts with plastid nucleoid protein pTAC5 (PLASTID TRANSCRIPTIONALLY ACTIVE5) and is required for normal chloroplast development in *Arabidopsis* under heat stress [67]. Chloroplast-localized molecular chaperones are crucial for heat stress responses, maintaining protein homeostasis within the organelle. For instance, CHLOROPLAST SIGNAL RECOGNITION PARTICLE 43 (cpSRP43) prevents the aggregation of tetrapyrrole biosynthesis (TBS) proteins and stabilizes them under heat stress, as demonstrated by Ji et al. [68]. The chloroplast-localized Hsp70CP1, an HSP70 family member in rice, is critical for both chloroplast development and protein homeostasis during high-temperature stress [69], emphasizing the importance of chloroplast HSPs in thermotolerance.

#### 2.3.4. Phytohormone and HSR

Phytohormones are a class of organic molecules that regulate plant growth, development, and responses to various environmental stimuli. Different phytohormones play crucial roles in plants’ adaptation to heat stress.

By maintaining photosynthetic efficiency, abscisic acid (ABA) enables *Arabidopsis* to recover from the detrimental effects of heat stress [70]. ABA-biosynthesis-*null* mutants exhibit reduced heat tolerance [71]. Specifically, the mutants *abi1*, *abi2*, and *uvh6* show the most severe defects in root growth and seedling survival under heat stress in *Arabidopsis* [72]. ABA enhances heat tolerance by increasing ROS levels and inducing the expression of NADPH oxidase-coding genes [73]. Additionally, ABA improves heat resistance by regulating the expression of *HSFs* and *HSPs*. For example, a deficiency in ABA biosynthesis at high temperatures leads to a reduced expression of HSP70 and oxidant-related genes mediated by GR245^DS^ [74].

Ethylene (ET) is also a crucial phytohormone in HSR. Heat treatment increases ethylene production, which subsequently elevates the expression of ethylene-responsive genes [75,76]. This, in turn, promotes heat tolerance [77]. Studies in tomatoes suggest that ET plays a role in ER stress and the UPR. The exogenous application of 1-aminocyclopropane-1-carboxylic acid (ACC), an ethylene precursor, upregulates UPR-related genes such as *SlIRE1*, *SlBiP*, and *SlbZIP60* [78,79].

Other phytohormones, including cytokinins (CK), BRs, and jasmonates (JAs), also play a role in regulating plant heat tolerance. Under heat stress, *Arabidopsis* leaves exhibited the downregulation of genes involved in CK biosynthesis, including *ISOPENTENYL TRANSFERASE 3* (*IPT3*), *IPT5*, and *IPT7*, while upregulating genes associated with CK degradation, such as *CYTOKININ OXIDASE 3* (*CKX3*) and *CKX6*. This indicates that heat stress reduces CK levels [80]. CK is hypothesized to negatively affect tobacco heat tolerance, as the ectopic expression of the *Arabidopsis CYTOKININ OXIDASE/DEHYDROGENASE 1* (*CKX1*) gene, which encodes a major CK-deactivating enzyme, in tobacco plants resulted in reduced heat tolerance [81,82]. BR also functions in HSR. Under heat stress, plants treated with brassinosteroids (BRs) exhibit two distinct gene regulation patterns: (1) BR restores the expression of developmental proteins that are inhibited by heat, and (2) BR enhances the production of protective proteins to levels exceeding those induced by heat stress alone [83]. These expression patterns suggest that BR improves heat tolerance. The exogenous application of methyl jasmonate (MeJA) has been demonstrated to significantly enhance the heat tolerance of perennial ryegrass [84]. This improvement is attributed to mechanisms such as osmotic adjustment, the activation of antioxidant defense systems, and the modulation of JA-responsive gene expression [85]. Additionally, JA is proposed to regulate stomatal apertures during heat stress and other combined stresses [86,87].

**Figure 1:**
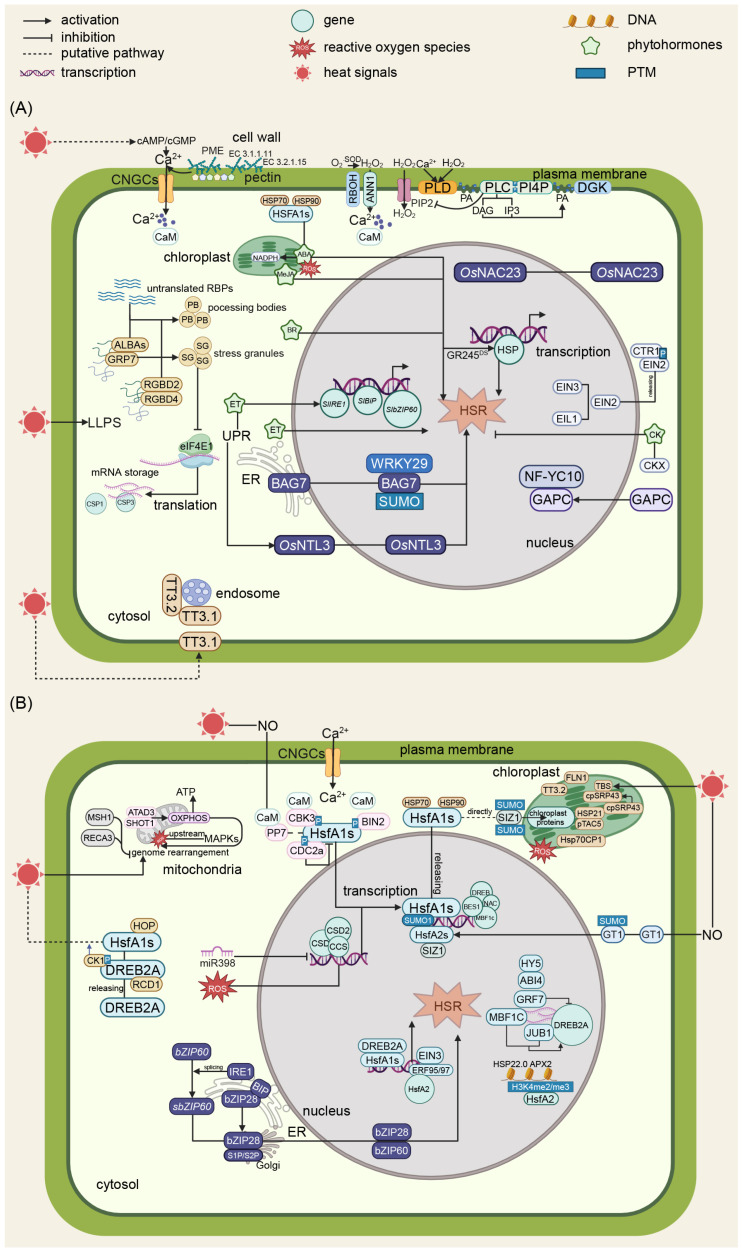
Schematic diagrams of the molecular regulation of HSR in plants. (**A**) A schematic diagram of calcium signaling and phytohormone regulation in plants’ heat stress responses. Upon exposure to heat stress, plant cells initiate a sophisticated signaling cascade beginning with membrane fluidity changes that activate calcium channels CNGCs and ANN1, leading to Ca^2+^ ion influx. CAM functions as a Ca^2+^ sensor. This triggers the production of ROS including hydrogen peroxide and superoxide anions, which function as secondary messengers. Phytohormones, such as ABA, ET, CK, BR, and JA, participate in diverse biological processes. ABA, BR, ET, and JA are positive regulators in plant heat tolerance, while CK is a negative regulator. (**B**) A schematic diagram of transcription regulation, UPR, and semiautonomous organelle regulation in plants’ heat stress response. CaM functions as Ca^2+^ sensors that bind with PP7 and CBK3, modulating HsfA1s’activity. SUMOylation mediated by SIZ1 and various phosphorylation events regulate the activity of key transcription factors. HsfA1s play a vital role in heat signal transduction that usually bind with HSP70 and HSP90. Released HsfA1s from the HsfA1s-HSP70/90 complex upon heat stress induce the transduction of stress-responsive genes, resulting in a heat stress response. Concurrently, HOP and BAG7 maintain proper protein folding, while heat stress causes ER stress, which leads to UPR through two crucial transcription factors, bZIP28 and bZIP60, that subsequently induce a heat stress response. Semiautonomous organelles like mitochondria and chloroplast are also critical components of plant heat stress. Within mitochondria, heat stress triggers ROS production and affects ATP synthesis, while GRP7 and other mitochondrial proteins contribute to stress signaling. These mitochondrial signals integrate with cytosolic pathways, influencing downstream responses. Chloroplasts contribute to Ca^2+^ and reactive oxygen species dynamics, highlighting the inter-organellar communication during stress responses. Negative regulators including ABI4 and RCD1 provide precise control over the heat stress response, ensuring appropriate cellular adaptation. Globes in different colors within characters represent distinct genes, proteins, or molecular compounds.

The heat stress signaling network is summarized in Figure 1.

### 2.4. Cold Stress Signal Perception

#### 2.4.1. Cold Stress Sensing via Membrane Fluidity

In plants, the cell membrane serves as the primary interface for both perceiving temperature signals and defending against cold stress [88]. Temperature directly and reversibly influences membrane fluidity, with cold-induced membrane rigidification recognized as an initial cold-sensing event [89]. Plants can modulate plasma membrane viscosity in response to moderate temperature fluctuations by adjusting the ratio of desaturated fatty acids, thereby mitigating temperature-induced alterations in plasma membrane morphology and function. Specifically, under cold conditions, an accumulation of desaturated fatty acids in cell membranes leads to a reduction in plasma membrane viscosity [90]. FATTY ACID DESATURASE 2 (FAD2) plays a crucial role in maintaining membrane fluidity; its dysfunction impairs plasma membrane viscosity homeostasis and diminishes cold tolerance [90]. Evidence suggests that membrane rigidity can induce the expression of cold stress response genes even at 25 °C, while increased membrane fluidity can repress these genes even at 4 °C [89,91]. Furthermore, cold-induced membrane rigidification activates the DIACYLGLYCEROL KINASE (DAGK) pathway, which subsequently promotes membrane fluidity and enhances cold resistance [92].

#### 2.4.2. Cold Stress Sensing via Membrane-Localized Protein Kinases

Membrane-localized proteins, including RECEPTOR-LIKE KINASES (RLKs) and HISTIDINE KINASES (HKs), are known to detect alterations in membrane fluidity, which in turn affects their conformation and activity [93,94]. In plants, multiple classes of RLKs play critical roles in modulating cold resistance. For instance, *Arabidopsis* calcium/calmodulin (CaM)-REGULATED RECEPTOR-LIKE KINASES (CRLKs) function as a bridge between calcium/calmodulin signaling and cold signaling pathways, thereby positively regulating cold resistance [95]. Another example is CRPK1 (cold-responsive protein kinase 1), a plasma membrane-localized kinase that phosphorylates the conserved phosphopeptide-binding protein 14-3-3. Upon phosphorylation, 14-3-3 translocates from the cytoplasm to the nucleus, where it interacts with C-REPEAT BINDING FACTOR (CBF), a key cold-responsive transcription factor, and promotes its proteasome-mediated degradation [96,97]. Furthermore, CTB4a (COLD TOLERANCE AT BOOTING STAGE), which encodes a LEUCINE-RICH REPEAT RECEPTOR-LIKE PROTEIN KINASE (LRR-RLK), confers varying degrees of cold tolerance in rice depending on the allele. CTB4a interacts with AtpB, the β-subunit of ATP synthase, enhancing ATP synthesis and thereby increasing cold tolerance specifically in the booting stage in rice [98].

Histidine kinases also contribute to cold stress responses. The HISTIDINE KINASE 33 (Hik33), found in *Synechocystis*, relies on membrane rigidity to perceive and respond to cold signals. It contains a P-type linker, a leucine zipper, and a PAS (P er A RNT S im) structural domain. Cold stress-induced changes in membrane fluidity trigger conformational changes in the P-type linker, leading to Hik33 activation through protein dimerization. This activation subsequently regulates the expression of numerous cold-inducible genes [99,100,101,102]. In *Arabidopsis*, the histidine kinases AHK2 and AHK3 (ARABIDOPSIS HISTIDINE KINASE 2 and ARABIDOPSIS HISTIDINE KINASE 3), which function as cytokinin receptors, negatively regulate the cold stress response, thus linking cytokinin signaling to cold stress pathways [103]. Specifically, AHK2 and AHK3 mediate the cold-induced expression of Type-A *ARABIDOPSIS RESPONSE REGULATORS* (*ARRs*) genes, which are known to be induced by cytokinins and to negatively regulate cytokinin functions [103,104]. While cold stress induces the expression of Type-A *ARR* genes, these genes act as negative regulators of the cold stress response. Notably, the cold-induced expression of Type-A *ARR* genes mediated by AHK2 and AHK3 is not associated with increased cytokinin levels during cold stress [103].

#### 2.4.3. Cold Stress Sensing via Ca^2+^

In plants, Ca^2+^ functions as crucial second messengers in response to environmental cues, with alterations in intracellular Ca^2+^ concentration representing an early event during cold stress. Exposure to cold stress rapidly triggers a significant elevation in cytoplasmic Ca^2+^ concentration ([Ca^2+^]_cyt_) [105,106,107,108]. Notably, prolonged or repeated cold exposure diminishes the magnitude of these [Ca^2+^]_cyt_ changes, suggesting that cold acclimation involves the modulation of calcium signaling pathways [105,109]. Several key proteins mediate Ca^2+^ influx during cold stress. CNGCs, widely distributed across cytoplasmic, nuclear, and ER membranes, are calcium-permeable cation channels [110] implicated in thermal perception, heat tolerance, and pathogen-associated molecular pattern-triggered immunity (PTI) through the mediation of Ca^2+^ influx [15,17,18,111]. Furthermore, CNGCs participate in cold stress responses. For example, in rice, the *Arabidopsis* OPEN STOMATAL 1 (OST1) homolog *Os*SAPK8 phosphorylates and activates *Os*CNGC9 under cold stress, initiating Ca^2+^ influx and downstream responses. Similarly, *Os*DREB1A enhances *OsCNGC9* expression, leading to increased Ca^2+^ influx and improved cold resistance [111,112]. MID1-COMPLEMENTING ACTIVITY 1 and 2 (MCA1 and MCA2), mechanosensitive plasma membrane proteins identified in *Arabidopsis thaliana*, also mediate calcium inward currents [113,114,115]. Cold stress induces MCA1- and MCA2-mediated calcium influx, resulting in a marked increase in [Ca^2+^]_cyt_ and regulating cold resistance via a CBF/DREB1-independent pathway [107]. CHILLING-TOLERANCE DIVERGENCE 1 (COLD1), a G-protein signaling regulator localized at both the plasma membrane and ER, represents a significant quantitative trait locus for cold resistance. In *Oryza rufipogon*, COLD1 interacts with RICE G-PROTEIN α SUBUNIT 1 (RGA1) to perceive cold signals and activate calcium channels, thereby facilitating calcium influx from the apoplast [116]. COLD1 also enhances the GTPase activity of RGA1, potentially shifting the equilibrium between GDP-bound and GTP-bound RGA1, leading to RGA1 self-activation [116,117]. Finally, OST1, a serine/threonine protein kinase central to ABA signal transduction, is activated by cold stress. Activated OST1 phosphorylates ANNEXIN1 (*At*ANN1), a plasma membrane-localized calcium transport protein, enhancing its calcium transport activity. This active form of *At*ANN1 mediates rapid Ca^2+^ influx, regulating the expression of *cold-regulated* (*COR*) genes and ultimately enhancing cold tolerance [118,119].

### 2.5. Cold Stress Signal Transduction in Plants

#### 2.5.1. Ca^2+^ Signaling

In plants, Ca^2+^ signaling plays a crucial role in mediating responses to both biotic and abiotic stresses, with plants decoding Ca^2+^ signatures to elicit specific gene expression patterns [120]. CALMODULIN-BINDING TRANSCRIPTION ACTIVATORS (CAMTAs) are key transcriptional regulators and Ca^2+^ sensors in this process. A proposed model suggests that CAMTA proteins detect cold-induced increases in Ca^2+^ concentration through interactions with calmodulin proteins. The activity of CAMTA proteins is modulated by both Ca^2+^ concentration and the strength of these interactions [121,122]. Specifically, 4Ca^2+^-CaM-CAMTA interactions amplify different Ca^2+^ signals non-linearly, enabling a differential decoding of Ca^2+^ signals and resulting in specific CAMTA-regulated gene expression responses [123]. Following the perception of temperature signals, plants activate relevant gene expressions primarily through protein kinases. A prominent class of Ca^2+^ sensors is the calcium-dependent protein kinase (CDPK), characterized by an N-terminal variable domain, a serine/threonine kinase domain, a conserved auto-inhibitory junction domain, and a C-terminal calmodulin-like domain. The C-terminal calmodulin-like domain is critical for recognizing changes in Ca^2+^ concentration and transducing signals, initiating downstream phosphorylation cascades [124,125,126]. For instance, cold stress activates *Os*CPK17, which in turn activates the water channel protein *Os*PIP2, leading to alterations in cell membrane permeability and osmoregulation [127]. Also in rice, under cold stress, *Os*CPK24 enhances cold tolerance in rice by increasing the levels of proline (Pro) and glutathione (GSH), with the increase in GSH levels correlating with the Ca^2+^-regulated thioltransferase activity of *Os*Grx10 [128]. Furthermore, in maize, COLD-RESPONSIVE OPERATION LOCUS 1 (COOL1), a bHLH transcription factor, is phosphorylated by CPK17 upon the cold-induced translocation of CPK17 to the nucleus. Stabilized COOL1 then negatively regulates cold tolerance in maize by repressing the expression of downstream *COR* genes, such as *DREB1* and *TREHALOSE-6-PHOSPHATE SYNTHASE* (*TPS*) [129]. Thus, the CPK17-COOL1 module acts as a regulatory brake, preventing the over-activation of stress responses and exhibiting a strong association with adaptive cold tolerance in high-latitude maize varieties [129]. Notably, while *At*CPK1, *Os*CDPK7, and *Os*CPK17 have been shown to improve cold tolerance in *Arabidopsis* and rice, they do not appear to be involved in the transcriptional regulation of certain key cold-induced genes [127,130,131]. In contrast to CDPKs, the CALCINEURIN B-LIKE (CBL) protein family and their interacting kinases (CIPKs) represent a more flexible combinatorial signaling model, with separate proteins responsible for Ca^2+^-binding and kinase activity, respectively [132,133]. For example, Calcineurin B-like interacting protein kinase 7 (*Os*CIPK7), a serine/threonine protein kinase, is activated by cold stress. A kinase domain point mutant of *Os*CIPK7 exhibits improved protein stability and kinase activity, thereby conferring enhanced cold resistance in rice [134].

#### 2.5.2. Other Messenger Molecules Involved in Cold Signal Transduction

Beyond Ca^2+^, other messenger molecules play crucial roles in mediating plant responses to cold stress. One prominent example involves ROS, including singlet oxygen (^1^O_2_), superoxide anions (O_2_^˙−^), hydroxyl radicals (OH^−^), and hydrogen peroxide (H_2_O_2_) [135,136,137], which are rapidly produced upon exposure to low temperatures. Specifically, hydrogen peroxide (H_2_O_2_) levels exhibit a dramatic increase in the initial stages of cold stress, followed by a rapid decline after approximately 8 h [138]. Correspondingly, antioxidant enzymes are activated to scavenge ROS. Catalase (CAT) functions as a primary defense mechanism during the first 2 h of cold stress, while ascorbate peroxidase (APX) assumes a more critical role in ROS regulation by the 4th hour. Superoxide dismutase (SOD) also contributes to ROS detoxification alongside CAT and APX [138]. Furthermore, to manage H_2_O_2_ accumulation under cold stress, plants activate a scavenging system that converts glutathione (GSH) to oxidized glutathione disulfide (GSSG) via glutathione reductase (GR) [139,140,141]. Nitric oxide (NO) also functions as a key signaling molecule in cold stress responses. Zhao et al. demonstrated that cold treatment upregulates the expression of *NITRATE REDUCTASE1* (*NIA1*), a gene involved in NO synthesis, leading to enhanced nitrate reductase (NR) activity and increased endogenous NO production in *Arabidopsis* leaves [142]. NO also influences the accumulation of proline (Pro), an important osmoprotectant that maintains cellular osmotic pressure and prevents dehydration. Specifically, NO transcriptionally promotes Pro synthesis and inhibits its degradation, thereby enhancing cold resistance [142,143,144,145]. However, NO’s role in cold stress is complex. Cantrel et al. showed that NO negatively regulates the formation of phytosphingosine phosphate and ceramide phosphate, two biologically active lipid molecules associated with cellular immunity, programmed cell death, and the maintenance of cellular homeostasis, suggesting that NO functions as an intermediate in cold and lipid signaling pathways [146,147,148]. In line with this, Costa-Broseta et al. (2018) [149] reported that NO negatively regulates cold tolerance in *Arabidopsis thaliana* by reducing the production of osmoprotective and antioxidant metabolites and altering hormone homeostasis. Conversely, Sehrawat et al. and Puyaubert et al. found that cold-induced NO production leads to the increased S-nitrosylation of superoxide dismutase (SOD), which promotes superoxide dismutation and ROS elimination [150,151]. These seemingly contradictory findings highlight the multifaceted role of NO in cold acclimation.

#### 2.5.3. C-Repeat/DREB Binding Factors (CBFs) Are Master Regulators in Plant Cold Responses

C-REPEAT/DREB BINDING FACTORS (CBFs) are core components in cold stress responses, belonging to the DREB subfamily A-1 of the ERF/APETALA2 transcription factor family. In *Arabidopsis*, four *CBF* genes exist. Specifically, *CBF1/DREB1B*, *CBF2/DREB1C*, and *CBF3/DREB1A* are induced by cold stress, while *CBF4* is not [152,153]. The functional importance of CBFs is further supported by studies showing that the heterologous expression of *CBF* genes enhances cold tolerance in various plant species [154,155]. Under cold stress, the expression of *CBF* genes is induced, and the resulting CBF proteins bind to CRT/DRE motifs, which are predominantly found in the promoter regions of *COR* genes [49,156,157,158,159,160]. This binding activates the expression of a subset of *COR* genes, thereby increasing cold tolerance [160]. Quantitative analyses suggest that CBFs regulate the expression of 10–20% of *COR* genes in *Arabidopsis* [161].

INDUCER OF CBF EXPRESSION 1 (ICE1), an MYC-like bHLH transcription factor, serves as a primary activator of *CBF* gene expression [162,163]. Under cold stress, ICE1 directly binds to the MYC element within the *CBF* gene promoter, initiating transcription [162,164]. However, this activation is subject to negative regulation. Mitogen-activated protein kinases MPK3 and MPK6 phosphorylate ICE1, reducing its transcriptional activity and promoting its degradation. This process negatively regulates the ICE1-CBF-*COR* cascade, ultimately diminishing cold tolerance [94,165,166,167]. Conversely, the MEKK1-MKK1/2-MPK4 cascade antagonizes the MPK3/6 pathway, contributing to improved cold tolerance [167]. Furthermore, under cold stress, OST1 phosphorylates ICE1 and inhibits the High Expression Of Osmotically Responsive Gene 1 (HOS1)-mediated ubiquitination and degradation of ICE1. MAPK3 sustains ICE1 phosphorylation, collectively enhancing ICE1 stability and transcriptional activity. This, in turn, increases *CBF* expression and cold tolerance [118,166]. The PP2C protein ABA-INSENSITIVE 1 (ABI1) negatively regulates OST1; phosphorylation upon ABI1 dissociation activates *ICE1* expression [118,168,169,170].

MYB15, a member of the MYB transcription factor family, also plays a regulatory role. Cold stress activates MPK3/6, leading to MYB15 phosphorylation, which reduces its DNA-binding affinity. Phosphorylated MYB15 dissociates from the *CBF* gene promoter, a prerequisite for ICE1 binding to the MYC recognition element and subsequent *CBF* gene activation [128,171,172]. Moreover, PUB25 and PUB26, homologous U-box E3 ubiquitin ligases, ubiquitinate MYB15, promoting its degradation. Under cold stress, OST1 specifically phosphorylates PUB25 and PUB26, enhancing their activity, which further promotes MYB15 degradation, *CBF* gene expression, and, ultimately, cold tolerance [173].

CAMTAs are another class of transcription factors involved in CBF regulation. These proteins contain calmodulin-binding structural domains that enable direct DNA binding and transcriptional activation [174]. The *CBF2* gene promoter contains several conserved DNA motifs (CMs), with CM2 identified as a key element positively regulating the response to cold stress [122]. CAMTA3 has been shown to be the primary protein binding to the CM2 element, positively regulating *CBF2* gene expression [122]. Furthermore, CAMTA1, CAMTA2, and CAMTA3 act synergistically to induce rapid *CBF* gene expression, influencing the expression levels of approximately 15% of genes induced by cold stress after 24 h [175].

*CBF* gene expression is also subject to regulation by circadian rhythms, which provides an adaptive advantage to plants facing diurnal and seasonal temperature fluctuations [176,177]. The core circadian oscillator CIRCADIAN CLOCK ASSOCIATED1/LATE ELONGATED HYPOCOTYL (CCA1/LHY) not only regulates the circadian rhythm of *CBF* gene expression but also responds to temperature decreases by strongly inducing *CBF2* and *CBF3* expression and weakly inducing *CBF1* expression, thereby enhancing cold tolerance [177,178]. PSEUDO RESPONSE REGULATORs (PRRs) are important components of the circadian regulatory mechanism. PRR9, PRR7, and PRR5 regulate the cyclic expression of stress-responsive genes, including *DREB1/CBF*, through circadian rhythms and time-dependent gating mechanisms [179]. In contrast, PHYTOCHROME-INTERACTING FACTOR 3 (PIF3) inhibits *CBF* expression under cold stress conditions, suggesting its role as an integrator of light and temperature signaling [180].

Phytohormone signaling pathways also exert control over *CBF* gene expression. Within the ethylene signaling pathway, key components ETR1 and ETR4 enhance cold tolerance, whereas EIN2 and EIN3/EIL1 inhibit it. EIN3 directly negatively regulates the expression of *CBF* and *ARR* genes in the cytokinin signal transduction pathway [181]. The jasmonate signaling repressor, JASMONATE ZIM-DOMAIN (JAZ), represses the transcriptional function of ICE1, while jasmonic acid positively regulates the ICE-CBF/DREB1 pathway, modulating cold resistance in *Arabidopsis* [182]. BR not only promotes plant growth but also enhances resistance to abiotic stresses, including cold stress [183,184]. BR regulates the transcriptional level or post-translational modifications of the bHLH transcription factor CES (C-TERMINAL ENCODED STEROL-REGULATED TRANSCRIPTION ACTIVATOR, CESTA) and its homologs BRASSINOSTEROID ENHANCED EXPRESSION 1 (BEE1) and BEE3, which in turn regulate the expression of *CBF* and *COR* genes, leading to improved cold resistance [184]. In the BR signaling pathway, Brassinosteroid insensitive 2 (BIN2) and its homologs BIL1 and BIL2 negatively regulate the expression of *Brassinazole-resistant 1* (*BZR1*) and its homolog *BES1* [185,186,187]. BIN2 negatively regulates the expression of *CBFs*. Cold stress induces the accumulation of dephosphorylated BZR1, which directly binds to the promoters of the *CBF1* and *CBF2* genes and positively regulates their expression [188].

The transcription factor ZINC FINGER OF ARABIDOPSIS THALIANA 12 (ZAT12) also plays a role in cold stress responses. The expression of *ZAT12* results in the accumulation of transcripts encoding arginine decarboxylase, an enzyme involved in the synthesis of the cold-resistant compound putrescine. However, constitutive *ZAT12* expression attenuates the activation of the *CBF1/2/3* genes under cold stress conditions, suggesting that ZAT12 negatively regulates the CBF-mediated cold stress response pathway [189]. The seemingly contradictory regulatory roles of ZAT12 in the response to cold stress in fact exemplify the complexity of the plant regulatory network.

Specific signaling molecules also regulate *CBF* expression. Under cold stress conditions, NO decreased *CBF1/3* expression, while it had no effect on *CBF2* transcript levels [148].

In contrast to transcriptional regulation, CBFs are also subject to post-translational modifications that primarily influence their stability. As previously mentioned, CRPK1 phosphorylates 14-3-3 proteins, promoting their nuclear localization where they interact with CBFs and trigger their degradation, thereby reducing CBF-mediated cold response and negatively regulating freezing tolerance [97]. Cold-activated OST1, however, phosphorylates BASIC TRANSCRIPTION FACTOR 3 (BTF3) proteins, which function as β subunits of the NASCENT POLYPEPTIDE-ASSOCIATED COMPLEX (NAC) and facilitate BTF3 proteins’ interaction with CBF proteins, as demonstrated in recent studies [190]. As a result, the stability of CBFs increases, thereby enhancing freezing tolerance.

#### 2.5.4. CBF-Independent Cold Signaling Pathways

Beyond CBF-mediated regulation, *COR* genes are also subject to control by a diverse array of other transcription factors [161,191]. For instance, ZAT12 directly regulates nine cold-induced and fifteen cold-repressed genes [189], and BZR1 influences cold stress responses by regulating not only *CBF* gene expression but also the expression of other *COR* genes independent of CBFs, such as *WRKY6*, *PYR1-LIKE 6* (*PYL6*), *SUPPRESSOR OF OVEREXPRESSION OF CO1* (*SOC1*), *JASMONIC ACID CARBOXYL METHYLTRANSFERASE* (*JMT*), and *SENESCENCE-ASSOCIATED GENE 21* (*SAG21*) [188]. In *Arabidopsis*, the salicylic acid (SA) signaling component NON-EXPRESSER OF PATHOGENESIS-RELATED GENES 1 (NPR1), well-established for its role in plant immunity, also participates in cold acclimation, acting independently of CBF1 [192]. Specifically, cold stress triggers NPR1 monomerization and its subsequent translocation to the nucleus [192]. Within the nucleus, NPR1 interacts with Heat Shock Factor A1 (HSFA1) transcription factors, as well as potentially other transcription factors, to enhance the expression of cold-induced, heat stress-responsive genes, thereby promoting cold acclimation. Given that HsfA1s are master regulators in plant heat responses, the diverse roles of NPR1 and HsfA1 in mediating multiple stress responses merit further investigation to fully elucidate their functions [192]. Furthermore, the presence of numerous CRT/DRE motifs in the promoters of *COR* genes suggests a role for calcium signaling in the response to cold stress, a hypothesis supported by the observation that Ca^2+^ regulates these motifs [193]. CAMTAs can also modulate cold response, either by promoting *CBF* gene expression or by directly regulating *COR* gene expression independently of CBF [175]. The *Arabidopsis ces-D* mutant, deficient in CES, exhibits significantly reduced cold resistance [184]. Notably, in *ces-D*, the expression of 269 genes outside the CBF regulatory network is more strongly induced, while 298 genes are more strongly repressed, indicating that CES regulates *COR* gene expression both through CBF-dependent and CBF-independent pathways, particularly those involved in fatty acid and lipid biosynthesis and metabolism [184].

Post-transcriptional regulation represents another critical layer of control affecting *COR* gene function. For example, STA1 (STABILIZED1), a PRP6-like splicing factor encoding a cold-induced nuclear protein, plays a significant role in plant responses to abiotic stresses [194]. STA1 functions both as a pre-mRNA splicing factor in mRNA splicing and in the turnover of unstable transcripts [194]. RCF1 (REGULATOR OF CBF GENE EXPRESSION 1), a cold-inducible DEAD (Asp-Glu-Ala-Asp) box RNA helicase, is essential for ensuring accurate pre-mRNA splicing and acts as a key positive regulator of cold tolerance [195].

#### 2.5.5. Semiautonomous Organelles and Cold Stress

Cold stress impacts both the structure and function of chloroplasts, which, in turn, serve as sensors for cold stress and mediate the plant’s response to it. Cold stress alters the abundance of proteins involved in photosynthesis. An iTRAQ-based proteomic analysis of chilling-tolerant and chilling-sensitive rice lines revealed the dynamic response of chloroplast photosynthetic proteins to chilling conditions, suggesting that certain chloroplast photosynthetic proteins may contribute to cold tolerance in rice [196,197]. Chilling stress modulates the redox state of chloroplasts, leading to the generation of ROS. In *Arabidopsis*, the regulation of chloroplast-to-nucleus ROS signaling serves as a strategy to enhance plant acclimation to cold stress [197,198]. ROS is also revealed to promote SA production during cold stress. *Arabidopsis* FILAMENTOUS TEMPERATURE-SENSITIVE H5/YELLOW VARIEGATED1 (FtsH5/VAR1) is a thylakoid-localized, ATP-dependent zinc metalloprotease that positively regulates cold tolerance [199]. The *var1-1* mutant exhibits a pronounced chlorotic/variegated phenotype and stunted growth under cold stress, which is attributed to excessive SA production induced by EXECUTOR1 and EXECUTOR2 (EX1 and EX2)-mediated ROS bursts [199]. In addition, chloroplast translational function is important for chilling tolerance. RNA-BINDING DOMAIN 1 (RBD1) is one of the four RNA-binding proteins localized to chloroplasts [200]. RBD1 is expressed in green tissues and localizes to the chloroplast nucleoid [200]. It directly binds to 23S rRNA, and RBD1 exhibits a stronger binding affinity under chilling conditions than at normal temperature [200]. This temperature-dependent binding affinity likely explains the observed mutant phenotypes [200]. Specifically, *rbd1* mutants fail to generate mature 23S rRNAs and show impaired 23S rRNA processing and chloroplast translation capacity [200]. This study establishes RBD1 as a regulator of 23S rRNA processing while demonstrating the importance of chloroplast translational function for chilling tolerance. The role of mitochondria in responses to cold stress remains largely unclear.

#### 2.5.6. Phytohormone and Cold Stress Responses

Auxin is a pivotal regulator of DNA-damage-triggered selective cell death in plants under cold stress conditions. It has been established that exposure to low temperature can result in the induction of DNA damage within root stem cells and their daughter cells. This, in turn, leads to the re-establishment of an auxin maximum in the quiescent center, thus preventing the further division of root stem cells [201,202].

Gibberellin (GA) has been demonstrated to promote significant processes in plant growth and development. The exposure of *Arabidopsis* seedlings to cold stress has been shown to trigger a decrease in bioactive GA, which has been demonstrated to promote DELLA protein accumulation and lead to DELLA-mediated growth inhibition. The *Arabidopsis della* mutant has been shown to have significantly reduced survival under cold stress conditions [203].

The role of the cytokinin signaling pathway in cold signaling and cold stress-mediated adaptation mechanisms has also been well-documented [103,204,205]. In *Arabidopsis*, cold stress has been shown to induce the transcription of *CYTOKININ RESPONSE FACTORS* (*CRFs*) [206,207]. The *crf4* mutant exhibited heightened sensitivity to cold stress in comparison to *CRF4*-overexpressing plants [206]. It has been demonstrated that both cytokinin and temperature stress alter the proteome, with a significant portion of their co-regulatory proteins showing overlap [208]. The present study investigates the role of the *CYTOKININ RESPONSE REGULATOR* (*CRR*) in mediating crosstalk between ethylene and cytokinin in response to cold stress, with a view to elucidating its function in conferring cold tolerance in *Arabidopsis*.

ET signaling negatively regulates cold resistance by repressing the expression of *CBFs* and Type-A *ARABIDOPSIS RESPONSE REGULATORS* (*ARRs*) in *Arabidopsis* [181]. An affirmative correlation has been demonstrated between fluctuations in ET release and the capacity of plants to withstand low temperatures [209].

ABA has been shown to play a central role in plant abiotic stress tolerance [210]. It has been demonstrated that ABA accumulates gradually in response to low temperature, and that the exogenous application of ABA enhances cold tolerance in plants [211,212]. Furthermore, it has been shown that ABA improves *CBF* and *ICE1* gene expression levels [211].

BRs are a class of steroidal phytohormones that protect plants against various abiotic stresses [213]. An analysis of gene expression levels in different BR signaling mutants has shown that BRs enhance cold tolerance in plants, at least in part, by activating the CBF-*COR* pathway, as previously mentioned [183,184,188]. Exogenous treatment with appropriate concentrations of the most biologically active BR brassinolide (BL) enhances cold tolerance in *Arabidopsis* seedlings [183].

Jasmonate ZIM-domain (JAZ) proteins function as repressors of JA signaling, directly interacting with and repressing the activity of ICE1 [182]. JA accumulates during periods of cold, leading to the degradation of JAZ, which in turn promotes the expression of *CBFs* and consequently enhances the cold tolerance of plants [182].

Salicylic acid (SA), a pivotal growth regulator, has been demonstrated to modulate the physiological and biochemical properties of plants under abiotic stress conditions [214,215]. Furthermore, plants of *Cucumis sativus* L., maize, and tomato exhibited enhanced tolerance to cold stress following the application of exogenous SA [216,217,218,219].

Strigolactones (SLs) are carotenoid-derived phytohormones. It has been demonstrated that mutants which are deficient in solanum lactone biosynthesis and signaling demonstrate sensitivity to cold treatment. The exogenous application of GR24^5DS^ (strigolactone analog) has been demonstrated to enhance cold tolerance in both wild-type plants and strigolactone-deficient mutants [220].

**Figure 2:**
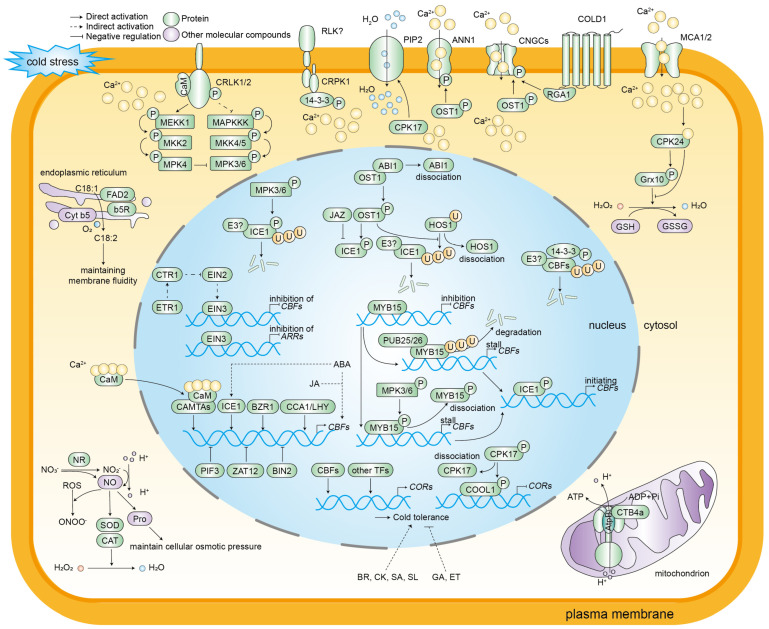
A schematic diagram of the molecular regulation of cold stress responses in plants. Cold stress induces alterations in cell membrane fluidity, consequently resulting in changes in the conformation and activity of the membrane-localized proteins RLKs and HKs. Calcium ions have been identified as second messengers in plants in response to environmental changes. Low temperature activates calcium channels, including CNGCs, MCA1/2, and ANN1. This activation results in the flow of Ca^2+^ from the ectoplasm to the cytoplasm and an increase in the concentration of Ca^2+^ in the cytoplasm. The subsequent consequence of this increase is the activation of a large number of Ca^2+^-related protein kinases. CaM and CAMTAs are pivotal proteins that sense changes in the concentration of Ca^2+^ and decode Ca^2+^ signals. CDPKs and CIPKs-CBL convey calcium ion signaling and activate cold stress-responsive gene expression. In addition to calcium ions, certain molecular compounds, including ROS and NO, play a regulatory role in the response of plants to cold stress. CBFs function as the core components of this response, with their expression levels being modulated by various transcription factors, such as CAMTAs, ICE1, MYB15, CCA1/LHY, 14-3-3, EIN3, BIN2, PIF3, and ZAT12. ICE1 directly binds to the *CBF* promoter to activate gene expression. The activity of ICE1 is positively regulated by OST1 and negatively regulated by JAZ, ABI1, and HOS1. The expression of the *COR* genes is contingent on the regulation of CBFs, and is further regulated by CDPKs and CIPKs-CBL, which activate cold stress-responsive genes. In addition, the *COR* genes are subject to regulation by transcription factors other than CBFs. Different phytohormones mediate plant cold stress responses. ABA and JA promote the expression of *CBF* genes, while ABA also activates *ICE1* expression. Additionally, BR, CK, SA, and SL enhance cold tolerance, while GA and ET negatively regulate cold resistance.

The cold stress signaling network is summarized in Figure 2.

## 3. Discussion

In this review, heat and cold responses are often discussed separately; however, plants in natural environments typically encounter complex conditions involving multiple simultaneous stresses. By comparing plant heat and cold perception and responses, several common features can be identified. For both heat and cold extremes, changes in membrane fluidity serve as a key mechanism, although they induce opposite effects: high temperatures increase fluidity, while low temperatures decrease it [221]. Under these stress conditions, extreme temperatures alter membrane fluidity, which in turn affects the activity of membrane-localized proteins, leading to Ca^2+^ influx or ROS generation. An intriguing example demonstrates that the membrane-localized proteins *Os*CNGC14 and *Os*CNGC16 function as temperature switches, enhancing tolerance to both heat and chilling stress [14]. Additionally, lipids and their metabolic enzymes play crucial roles in sensing both heat and cold stress signals. In terms of extreme-temperature responses, several common features are also evident. Transcriptional regulation is essential for both heat and cold responses. However, different master regulators are involved: HsfA1s for heat stress responses and CBF1 for cold responses. Both transcription factors are intricately regulated at the transcriptional and post-translational levels, initiating multiple downstream responses to mitigate the effects of extreme temperatures. Interestingly, some common components simultaneously regulate responses to both cold and heat stress. As discussed earlier, NPR1 may be involved in both cold and heat stress responses [192]. Identifying common mechanisms that enable plants to tolerate a broad range of stresses will be a worthwhile endeavor in the future. While the function of NPR1 suggests that some common components may be involved in both heat and cold stress responses, it is intriguing to investigate how plants prioritize concurrent heat and cold signals. Do heat-resistant plants exhibit compromised cold resistance, or vice versa? This question merits further investigation.

Although plant responses to extreme temperatures have been extensively studied in recent years, offering a relatively comprehensive understanding of the signaling networks involved in heat and cold stress responses, the mechanisms by which plants detect these temperature extremes remain largely unknown. Identifying additional temperature sensors is essential to filling this significant gap in our knowledge.

Semiautonomous organelles, such as mitochondria and chloroplasts, play critical roles in the perception and response to heat and cold stress. However, our understanding of their overall function remains fragmented. Future research should focus on elucidating the mitochondria/chloroplast-nucleus retrograde signaling pathways and the communication mechanisms between these semiautonomous organelles, which are of paramount importance.

Extreme temperatures pose a significant threat to crop productivity and food security. However, research on crop responses to heat and cold stress has lagged behind studies in *Arabidopsis*. Further investigation into temperature signaling networks in crops is crucial for directly supporting breeding efforts.

## Data Availability

No new data were created or analyzed in this study.
